
JOURNAL INFORMATION
==============================
NLM Title Abbreviation: ScientificWorldJournal
Journal ID: ScientificWorldJournal
NLM Journal ID: 101131163
Journal ID: TSWJ

The Scientific World Journal

ISSN: 2356-6140
EISSN: 1537-744X
Publisher: Wiley

ARTICLE INFORMATION
==============================
PMCID: PMC6084420
DOI: 10.1100/tsw.2001.21
Article ID: 594910
Article version: 1
Subjects: Announcement

Announcement of Ecological Modeling, a Distance Learning Course for the International Science Community

Jorgensen Sven Erik *
Institute A, Section Environmental Chemistry, Universty Park 2, 2100 Copenhagen Ø, Denmark
*Sven Erik Jorgensen: sej@dfh.dk
Publication date: 2001
Electronic publication date: 2001 Apr 4
Volume: 1
First page: 146
Last page: 147
Copyright: Copyright © 2001 Sven Erik Jorgensen.
Copyright year: 2001
License: This is an open access article distributed under the Creative Commons Attribution License, which permits unrestricted use, distribution, and reproduction in any medium, provided the original work is properly cited.
License URL: https://creativecommons.org/licenses/by/3.0/

==============================
Announcement
*TheScientificWorld* (2001) 1, 146–147
ISSN 1532-2246; DOI 10.1100/tsw.2001.21

TheScientificWorld
www.thescientificworld.com

### Announcement — *Ecological Modeling*, a Distance Learning Course for the International Science Community

Sven Erik Jørgensen*

*Institute A, Section Environmental Chemistry, University Park 2, 2100 Copenhagen Ø, Denmark; Fax: (45) 35306001*

**KEY WORDS:** distance learning, environmental models, parameters in ecotoxicology, ecotoxicology models, biogeochemical models, food chain models, eutrophication models, energy circuit language, ecosystems and communities, environmental toxicology, population dynamics models

**DOMAINS**: ecosystems and communities; environmental toxicology; environmental chemistry; environmental modeling

An internet-based distance learning course on Ecological Modeling[1] is available to provide an introduction to environmental science modeling. Broadly, the topics will cover:

- Modeling as a Tool in Environmental Management and Research
- Eutrophication and Food Chain Models
- Ecotoxicological Models

The course is suitable for all those with an interest in gaining a sound knowledge of modeling environmental, ecotoxicological, and ecological data, including researchers within academic institutions and industry, environmental consultants, and environmental managers around the world.

The course commences on the 1st April 2001, although students can join the course at any date thereafter. Course participants can work at their own pace and are assisted by the Course Tutor, S.E. Jørgensen of the University of Copenhagen, Denmark.

The course will consist of eight steps corresponding to chapters in the textbook *Fundamentals of Ecological Modeling*[2]. A third edition of the book will be published during the summer of 2001. In addition to the exercises and problems within the textbook chapters, each step will consist of additional exercises and a stepwise development of participants' own models. The results of exercises and the model development will be reviewed and commented by the Course Tutor. The eight steps and corresponding chapters within *Fundamentals of Ecological Modeling* (3rd ed.) are:

1. Chapters 1 + 2. *Introduction and How to go about Modeling.* The database, the background problem, and the conceptual diagram of the participant's own model is discussed.
2. Chapter 3. *Sub-Models and Process Descriptions*. The equations for the processes in participant's own model are presented and discussed.
3. Chapter 4. *Conceptual Models*. The conceptual diagram is discussed again after the processes have been expressed by equations.
4. Chapter 5. *Steady State Models*. The steady state edition of participant's own model is discussed, possibly by use of ECOPATH[3].
5. Chapter 6. *Population Dynamic Models*. It is presumed that the participant's own model is a dynamic biogeochemical model, but there will be occasion to use a software based on H.T. Odum's energy circuit language applied to a food chain[4]. The software is self-instructive and facilitates self-testing.
6. Chapter 7. *Biogeochemical Models*. The participant's own model is calibrated and validated. Three software testing eutrophication models can be used for model training.
7. Chapter 8. *Ecotoxicological Models*. Software denoted WINTOX[5] is used to estimate parameters for ecotoxicological parameters. Five additional exercises in the use of the software are applied.

* E-mail: sej@dfh.dk
© 2001 with author.

8. Chapter 9. *Recent Development in Ecological Modeling.* Final discussions on model results, including discussion on the suitability of one of the new approaches presented in Chapter 9 used for problem solving.

A course fee applies, which includes textbook, five software packages (three eutrophication models, a self-instructive software based on H.T. Odum's 'Energy circuit language applied to food chains', and WINTOX applied to the estimation of ecotoxicological parameters). Textbook and software will be sent at the beginning of course. The course can also provide STELLA[6] software, which is recommended for all small and medium size models, using small and medium-sized databases. STELLA is used extensively in the textbook to give illustrative modeling examples. Participants are also offered discounts on modeling books published by Lewis Publishers, U.S.

All interested in joining the course should send an e-mail to Professor Dr. Eng. Dr. Scient. S.E. Jørgensen at sej@dfh.dk. See also the course web site at www.enviromod.subnet.dk.

### REFERENCES

1. Internet course on Ecological Modelling, 2001: URL: www.enviromod.subnet.dk.
2. Jørgensen , S.E. (2001) *Fundamentals of Ecological Modeling*. Elsevier Science (in press).
3. ECOPATH 2000. Software. Published by Villy Christensen and David Pauly, Vancouver. URL: www.enviromod.subnet.dk.
4. Odum, H.T. Software, 1988 Software based on 'Energy circuit language applied to food chains' and published by Cathy Ewells
5. WINTOX 1997. Software published by S.E. Jørgensen, Bent Halling Sørensen og Henrik Mahler
6. STELLA 2000. Version 5.1. Software published by High Performance LTD.

Published: April 4, 2001
